# Educational Policies Matter: How Schooling Strategies Influence Refugee Adolescents' School Participation in Lower Secondary Education in Germany

**DOI:** 10.3389/fsoc.2022.842543

**Published:** 2022-06-22

**Authors:** Gisela Will, Regina Becker, Oliver Winkler

**Affiliations:** ^1^Leibniz Institute for Educational Trajectories (LIfBi), Bamberg, Germany; ^2^Martin Luther University Halle-Wittenberg, Halle, Germany

**Keywords:** educational policies, schooling strategies, refugees, school participation, newcomer classes, Germany

## Abstract

This article investigates the educational participation of refugee adolescents in Germany as a main European destination country of refugee migration. Opportunities and restrictions for school participation vary not only across countries–, but in the case of the Federal Republic of Germany, also within countries. The influence of different regional educational policies on refugees' educational participation and the extent to which they limit or enable individual agency, are however, widely understudied. We thus aim to analyze how different regional educational policies within Germany influence refugee students' educational participation regarding four central indicators: the duration until school enrollment, the type of class attended (newcomer vs. regular class), the type of school attended, and whether they are enrolled in settings appropriate for their age. We rely on a theoretical model which sees educational decisions as the result of rational cost-benefit calculations. The individual educational investments depend on individual motivations and resources within a given opportunity structure. We integrate the legal regulations via the opportunity structures into the theoretical model. Our analyses are based on data from 2,415 adolescents who were interviewed in the “ReGES–Refugees in the German Educational System” study. Our results show significant correlations between different regional educational policies and the four domains of educational participation. These effects remain stable when considering family and individual resources, as well as further control variables that previous research on social and ethnic educational inequality has shown to be relevant. Family and individual resources only partially influence educational participation. This indicates that refugee students and their parents have only limited options for action concerning their educational participation. Thus, our study shows that educational policies in fact matter: the assignment to a federal state plays a significant role in determining the duration until school enrollment, whether one is placed to a grade level age-appropriately, and whether one attends a newcomer class. Most significantly, legal regulations strongly influence refugees' chances of attending a higher school track (*Gymnasium*). Due to the low permeability of the German education system, this creates path dependencies for the further education and career paths of new immigrant students.

## Introduction

The number of refugees worldwide remains at a very high level. By the end of 2020, 82.4 million people worldwide were forcibly displaced (United Nations High Commissioner for Refugees (UNHCR), 2021). While the majority of refugees find refuge in countries of the Global South (in 2015, for example, this concerned nearly 86% of all refugees registered worldwide; see Oltmer, 2016), the number of refugees applying for asylum in the Global North increased in the mid-2010's. During the major refugee migration movement of 2013–2017, ~4.3 million refugees came to Europe, ~1.8 million of whom came to Germany (Bundesamt für Migration und Flüchtlinge (BAMF), 2018). Of the ~1.8 million refugees who arrived in Germany between 2013 and 2017, more than 30% were minors. For this group, integration into the educational system is a central prerequisite for inclusion in other areas of the arriving society in general, especially for later successful participation in the labor market. On the other hand, the large number of school-age refugees offer researchers the possibility to observe the integration of refugee children and adolescents into educational institutions in quantitative terms.

Generally, educational trajectories are strongly institutionally shaped, especially by educational policies such as education acts and school regulations, which (among others) define the timing of school entrance, duration until enrollment, and conditions for a student's assignment into a certain type of school (Mayer, 2004). Thus, on the one hand, educational policies enable individual scope for action, but on the other hand they can restrict individual opportunities for decision-making, and thus limit the individual's capacity to gain control over their educational trajectory. Especially at school, institutional control regarding educational transitions is particularly high, and individuals have limited agency in selecting their pathways (Giddens, 1984; Heinz, 1996).

Also, for newly immigrated refugee youths in Germany, educational participation in the general educational system is strongly regulated. These regulations can support or hinder the integration of newcomers into the educational system. Such institutional control over schooling is fixed within educational policies. In our study we therefore take a closer look at these educational policies and examine whether they are related to various important characteristics of educational participation. Our general research question is: *how do varying educational policies in Germany affect the educational participation of refugee students in lower secondary education?* To analyze the effects of different educational policies on educational placement, we build on theoretical models which understand educational decisions as the result of rational cost-benefit calculations (e.g., Breen and Goldthorpe, 1997). For a more detailed formulation of the theoretical model and our hypotheses, we consider in particular the assumptions of the life course approach. We consider the following four outcome variables:

1. Duration until school enrollment in Germany: the longer schooling is interrupted, the more likely it is that students' educational development will be impeded. Since many of the young refugees might have already have experienced long breaks in schooling due to the situation in their origin countries and the flight itself, it is important to integrate them into schools in the destination country as quickly as possible. Legal regulations on the concrete implementation of compulsory schooling for newly arrived children and adolescents can influence the time until school enrollment. In our study, we examine whether these regulations are actually related to the duration of school enrollment.
2. Type of class attended: the question of how newly immigrated children and adolescents, who do not speak the language of the destination country, should (initially) be schooled–the extreme poles vary between separate schooling, and teaching in regular classes as quickly as possible–has been around for a long time in Germany. This important question is highly controversial (see e.g., Reich, 2017) and has not yet been satisfactorily clarified because concerning their medium- and long-term effects on language acquisition, school participation, and labor market success of immigrant students separate schooling vs. teaching in regular classes have rarely been compared. It is also not clear how school education is actually organized, and to what extent given regulations can be implemented on the ground. In our study, we take a first step in approaching these questions. We examine how the education of newcomers is organized, specifically whether newcomers attend separate classes or not, and to what extent this organization is related to education policy regulations.
3. Access to different types of schools: Germany has a highly differentiated secondary school system. Upon entering in secondary education, students are on average 10 years old, i.e., tracking starts at an early age. The type of school attended largely determines which final qualifications can be achieved, and thus also significantly structures access to the labor market, since in Germany, educational qualifications and certificates are essential for labor market success (Bol and van de Werfhorst, 2011). Equal access to the different types of schools for newcomers entering secondary education at a later stage, and the role that educational policies play in this therefore constitute central issues. We examine which regulations concerning access to different school types for non-German speaking lateral entrants exist in each federal state and how these regulations relate to the actual school attendance of refugees.
4. Age-appropriate placement in a school class: new immigrants who do not have sufficient command of the language of instruction are often enrolled in classes in which the average age of students is much lower than their own. While interrupted school careers and a lack of knowledge of the language of the destination country make the assignment of refugees students to lower school grades appear necessary, it is unclear however, to what extent an age-inappropriate placement might have disadvantages for the social integration or the further life courses (e.g., entry into the labor market) of the students. Those whose age is higher than their class average could also be more likely to experience educational disengagement, alienation from school, or school detachment. On the other hand, older refugees who are still of school age but only have a few years of compulsory schooling left have fewer learning opportunities (i.e., time) at school, e.g., for acquiring German language skills. In this context, integration into a school class in which the average age is significantly lower than the refugees' own age can also be beneficial, as it enables the refugees to attend school for longer. We first consider which regulations on the allocation of individuals to class grades exist in each federal state, and second, analyze whether there is a connection between these educational policy regulations and age-appropriate placement.

Our analyses are based on a novel dataset “Refugees in the German Educational System (ReGES)” (Will et al., 2021) of 2,415 young adolescents aged between 14 and 16 at the time of the sample selection who were still in lower secondary education in the German general school system. Our results are not only important for politics and practice in Germany, but can also be relevant for all countries that accept newly arrived immigrants and want to integrate them successfully into their school systems–especially in the European Union where member states are obliged to guarantee the educational integration of refugee minors. The topicality and relevance of our findings is particularly evident because of the war in Ukraine in 2022. In this current situation, many school-age children and adolescents have had to flee Ukraine and seek protection in other countries.

We proceed as follows: after presenting some general information about the German school system, we introduce the relevant legal requirements for educational policies in Germany, and the level at which these legal requirements are implemented. We then present our theoretical model, which explains individual education decisions, and at the same time considers the educational policy regulations. In the next step, we apply this theoretical model to our four outcome variables, outline the state of research, and derive our assumptions “Theoretical Assumptions and State of the Research”. In “Data and Methods”, we describe the data which our analyses are based upon, the analysis strategies employed, and the operationalization of the variables used in our models. In “Results”, we report the results of our descriptive and multivariate analyses for each variable separately. In the “Discussion”, we highlight the implications of our research for practice and politics, as well as suggest areas for further research, and discuss the transferability of our results. In this context, we also address the limitations of our research.

## Theoretical Assumptions and State of the Research

### The German Context

In Germany, the educational system is not regulated nationwide, but the federal states determine over education. Nevertheless, there are some school system characteristics that are similar in all federal states. For example, the German school system features a relatively early tracking into different educational pathways. This usually happens after the fourth grade (in just three federal states after the sixth grade), which, in an international comparison, is very early in the students' educational careers.

From lower secondary education onwards, the school system in Germany is highly structured. There are three main educational paths. In addition to the *Gymnasium* (higher secondary track), which primarily prepares students for university, there are two less demanding types of school: the *Hauptschule* (lower secondary schools), which prepares students for vocational training; and the *Realschule* (middle secondary schools), which enables students to access more demanding options for vocational training. In the recent past, however, there has also been a growing share of schools that offer several educational tracks, such as *Gemeinschaftsschulen (*joint schools), which feature all three tracks, or those which offer only the intermediate and lower tracks (comprehensive schools). Despite increasing institutional differentiation and options for obtaining an upper secondary school degree (*Abitur*) at comprehensive schools, transition rates to upper secondary education are much higher in the *Gymnasium* than in comprehensive schools, which mainly lead to lower or intermediate school degrees (Statistisches Bundesamt, 2021a, Table 3.7). Especially immigrant youths in Germany are less likely to transition to *Gymnasium* after having completed a comprehensive school (Kurz and Böhner-Taute, 2016). Therefore, gaining access to the *Gymnasium* is still key to social upward mobility via education.

In contrast to the descendants of immigrants who are generally not evenly distributed across Germany, but tend to cluster in larger metropolitan areas in West German federal states, refugee children are more evenly distributed throughout Germany. This is due to the distribution according to the Königstein key (*Königsteiner Schlüssel*), whereby the admission quotas are calculated every year on the basis of tax revenue and the population size of the federal states. As a consequence, this means that all federal states and almost all municipalities have to deal with the question of how newly immigrated students with possibly insufficient German language skills can be integrated into school. Concerning schooling for newly immigrated students, including refugees, the federal states pass their own laws and regulations but they must incorporate the requirements set by international law (e.g., UN Convention on the Rights of the Child), European law, and the directives of the Standing Conference of the Ministers of Education and Cultural Affairs of the Länder in the Federal Republic of Germany (*Kultusministerkonferenz*)^1^ into their policies. Nevertheless, policies regarding the integration of newly immigrated students into the school system vary greatly from state to state^2^.

In our study, we describe and compare the educational regulations of five of the 16 federal states in Germany: Bavaria, Hamburg, North Rhine-Westphalia, Rhineland-Palatinate, and Saxony. The main reason for selecting these five federal states was because they feature the greatest amount of difference concerning their schooling strategies for newly arrived immigrants (for more details on the selection of the five federal states, see Will et al., 2018; Steinhauer et al., 2019). Regulations that relate to the school integration of refugee students particularly affect the timing of schooling and separate learning. We hypothesize that different configurations of these regulations between federal states affect four domains of educational participation of refugee youth (i.e., duration until school enrollment, type of class attended, attended school type, age-appropriate placement).

### Opportunity Structure and Educational Decisions

When examining educational participation, theoretical models are often used which see educational decisions as the result of rational cost-benefit calculations, in which individuals select the option that best suits their interests (Boudon, 1974; Erikson and Jonsson, 1996; Breen and Goldthorpe, 1997). The individual educational investments depend on individual motivations and resources within a given opportunity structure. Opportunity structures depend on institutions and norms, i.e., legal regulations, and constrain an individual's choice set. In the educational context, educational politics control, for example, the differentiation of school types, and set access conditions to different school tracks. Institutions thus create a set of opportunities (e.g., types of schools), but also formally limit access by setting restrictions or rules (e.g., grade point average). To pursue opportunities and meet requirements, individuals need to utilize their resources. In some instances, especially in the modern school system, institutions almost completely rule out individual choice and even force individuals to obey educational laws by using legal sanctions (e.g., enforcing compulsory schooling) (Kohli, 1985). We build on this theoretical perspective and integrate legal regulations for school attendance into the theoretical model via the opportunity structure, i.e., restrictions. We assume that school education for all school-age children and adolescents is prescribed by legal regulations, while (to some extent) agency is still provided.

Strong regulation particularly applies to two features of the German educational system that strongly restrict, channel, and set path dependencies for refugee students' school trajectories and school integration: the timing of enrollment, and separate schooling. Timing of enrollment and separate schooling can have standardizing effects on schooling careers (Brückner and Mayer, 2005) by producing temporally uniform school trajectories with minimized deviations. This standardization can vary between federal states. To some degree, education policies may also introduce de-standardization, i.e., multiple educational pathways at more dispersed ages and durations. Since education is controlled by federal states, regulations that impact (de-)standardization may vary between them. In the following, we will discuss the degree of standardization and the respective individual options for action for Germany in general in the two areas of timing of enrollment and separate schooling, before moving on to the specific regulations that prevail in the federal states that are part of our sample.

Currently, the timing of enrollment is largely standardized within federal states. During the 1980's, however, the German migration regime strongly favored return migration (Castles, 2006) and aimed to delay the migration of family members to Germany. Rapid enrollment in integrated classes with native students was therefore not the primary goal; instead, the focus was on separate classes and often even on instruction in the language of origin (see e.g., Karakayali et al., 2017). This was intended to make it as easy as possible for migrants to reintegrate into local school systems when they returned to their countries of origin. Already at that time, empirical studies pointed out that early access into the regular German school system would promote German language proficiency and chances for interethnic contacts for immigrant children (Esser, 1980, 1989), and an early enrollment date was later adopted in German politics as an integration strategy. At the present time, duration until school entry is regulated relatively similarly for all refugee children and adolescents across federal states. But even if all federal states favor early entry into school, regulations differ between federal states, and can create different opportunity structures for refugee families depending on where they live. We assume that there is hardly any room for individual decision-making. *We therefore seek to examine this in our analyses of our first dependent variable, duration until school enrollment. We investigate the influence of school policy regulations on the duration of enrollment as well as the (small) additional influence of the refugee families via individual resources*. However, timing of schooling is furthermore linked to age norms that are important for standardizing school entrance and exit (Kohli, 1985). But there is also some flexibility in this area. For example, children are normally enrolled into first grade between the age of 5–7, which offers parents and schools some possibilities to delay enrollment if they believe that the child might experience excessive demands (Faust, 2006). In this logic, many federal states place older refugee students in a lower school grade where other children are much younger but where the curriculum is less demanding. Although the educational system has introduced some degree of de-standardization here, we assume that this will increase the flexibility of schools in assigning individual students in particular, but not the room for maneuver of the individual students or parents themselves. *We test this in the models, in which we examine whether school policies are indeed a key determinant of schooling in an age-appropriate grade level, and to what extent refugee families can exert additional influence here*.

The second major area that structures the schooling of refugees is the question of separate or inclusive schooling. Upon entering the German school system, support measures for immigrant children largely involve schooling in separate learning environments. Separate newcomer classes are a classic instrument for the educational integration of immigrants, especially for teaching the German language. They were introduced in the mid-1960's in West-Germany (Helbig and Nikolai, 2015, p. 126) and experienced a “revival” during the large refugee influx (Brüggemann and Nikolai, 2016). Separate learning in newcomer classes as an effective educational policy for refugee students is highly controversial and subject to much debate (Karakayali et al., 2017). It has also not been implemented in all federal states in Germany. In fact, there is hardly any standardization in schooling between the individual federal states in Germany, and the concrete organization differs between federal states (Massumi et al., 2015). Some federal states strongly emphasize a single educational path for refugee students upon entering school, while others allow manifold pathways and also enable some degree of agency. *Therefore, we also consider the issue of separate schooling in a separate model, and analyze the extent to which school policies determine which refugees do or do not attend a separate newcomer class*. Some federal states have introduced newcomer classes in all types of schools, or also allow inclusive schooling in regular classes in all types of schools. Others provide newcomer classes solely in less demanding school types to permit more focused teaching. As the German school system is quite inflexible concerning educational upward mobility, especially for immigrant students (Kurz and Böhner-Taute, 2016), refugee students' opportunities for entering higher school tracks after attending newcomer classes in lower tracks may be diminished. *Thus, we address the question of the extent to which school policy rules have an impact on the type of school attended once adolescents attend a regular class*.

In summary, in the case of newly immigrated young people, we focus on legal regulations in the four domains of educational participation outlined above that relate to timing and separate schooling: the duration until school enrollment in Germany; the type of class attended (regular class vs. newcomer class); the type of school attended (academic track vs. other school form); as well as regarding whether students are enrolled in a suitable grade level for their age. In the next step, we will look in detail at the various indicators of participation in school education. In each case, we outline the state of the art in the research, present the federal regulations in these areas, and formulate our assumptions on the relationship of the regulations and educational integration.

### Educational Policies on the Schooling of Newly Arrived Immigrant Students

#### Duration Until School Enrollment

According to the European Reception Directive (Council Directive 2003/9/EC of 27 January 2003 Laying Down Minimum Standards for the Reception of Asylum Seekers, Art. 10), “member states shall grant minor children of asylum seekers and asylum seekers who are minors access to the educational system under similar conditions as nationals of the host member state.” Therefore, schooling is compulsory in Germany for all school-age refugees, including adolescents aged 14–16, who are the focus of our study. Additionally, compulsory education in Germany is not regulated nationwide, but by the school laws in each federal state. However, there is a minimum requirement of nine compulsory years of education in the general educational system in all federal states, and this is furthermore followed by compulsory vocational training in many federal states. Thus, it can be stated that the general schooling obligation still applies to all adolescents in our sample (for an overview of the regulations on compulsory schooling in different federal states, see Massumi et al., 2015).

However, the legal framework of the European Union on compulsory education allows institutions some degree of flexibility: according to the European Reception Directive (Council Directive 2003/9/EC of 27 January 2003 Laying Down Minimum Standards for the Reception of Asylum Seekers, Art. 10), access to the educational system shall not be postponed for more than 3 months after application for asylum, but it can be extended to 1 year in cases in which education is provided to facilitate the entrance to the educational system. Such institutional variation in compulsory schooling for refugees also exists between the German federal states (see Table 1 for an overview of the five federal states in our sample); in Hamburg, compulsory schooling starts upon arrival in the federal state, while in Bavaria, compulsory education starts 3 months after arrival in Germany. In North Rhine-Westphalia, Rhineland-Palatinate, and Saxony, compulsory schooling starts when refugees leave the reception center and are allocated to a municipality. However, there are indications that other aspects besides legal regulations influence the timing of school enrollment. For example, officials emphasize that a later school start helps to reduce the risk of having to later change school, enables a more permanent place of residence to be established beforehand, and avoids congestion of municipalities (Monitoring-Stelle, UN-Kinderrechtskonvention (DIMR), 2017). Such assumptions could suggest delays in school enrollment at the local or school level. Empirically, we found hardly any quantitative evidence on the average wait time for refugees before entering school. Qualitative studies, however, report that waiting times for school enrollment tend to be longer than administrative regulations normally admit (Lewek and Naber, 2017; Vogel and Stock, 2017; Münk and Scheiermann, 2020).

**Table 1:** Beginning of compulsory education.

**Federal state**	**Beginning compulsory education**
Bavaria	Three months after moving from abroad (Article 35 BayEUG)
Hamburg	Upon taking up residence in Hamburg (§37 HmbSG)
North Rhine-Westphalia	After assignment to a municipality (§34 SchulG NRW)
Rhineland-Palatinate	After assignment to a municipality (§56 SchulG RP)
Saxony	After assignment to a municipality (§§26, 28 SchulG SN; asylinfo.sachsen.de, 2021)

*Will and Homuth (2020); adapted and updated*.

As the assignment to a municipality could take considerably longer, especially *during times of high refugee immigration*, we assume that in states in which compulsory education starts only after refugee adolescents are assigned to a municipality, they have to wait longer until school enrollment. Overall, differences in duration until enrollment between federal states are likely to be observed. Families are not assumed to influence the waiting time to a large extent: we expect little effect of familial and individual resources, like parents' educational background or students' previous educational achievements on the time until enrollment^3^. However, analyses of asylum procedures in Germany show that the human and social capital of refugees does have an influence on the decisions on their asylum applications and the length of asylum procedures (see Kosyakova and Brücker, 2020), even if an examination of the legal regulations alone would not lead us to suspect such an influence. A shorter asylum procedure can subsequently lead to a faster assignment to a municipality, and thus to a faster start of a child's compulsory schooling. It is also possible that high parental education levels, as well as good school performance of the adolescents have a signaling effect, suggesting a quick and easy integration of the children into the school class. Thus, this can additionally accelerate processes in individual schools.

#### Type of Class Attended

In addition, we analyze whether the adolescents surveyed first attend a class for newly immigrated students, or whether they are directly enrolled in a regular class. The recast of the European Reception Directive (Council Directive 2013/33/EU of 26 June 2013 Laying Down Standards for the Reception of Applicants for International Protection (Recast), Art. 14) also requires member states to implement preparatory classes, including language classes that shall be provided to minors, if it is necessary, to facilitate their access to and participation in the educational system. Preparatory classes are separate classes for recently migrated students (also “welcome classes” or “newcomer classes”) who aim to learn the language of instruction. There are also partially integrative models where some school subjects are taught in regular classes together with students who were born in Germany, or at least have lived in Germany for a longer time.

Empirical findings indicate that separate schooling of refugee students is currently an important method of instruction in Germany. According to a non-representative survey of headmasters in four German federal states^4^, one half of all schools offered newcomer classes to refugee students at the start of school in Germany in 2017 (Hofherr, 2020). Regarding the students themselves, a representative survey of refugees residing in Germany found that one third of school-age refugee students attended a newcomer class in 2016 (IAB-BAMF-SOEP) (de Paiva Lareiro, 2019). Focusing on the age group relevant to our study, the IAB-BAMF-SOEP survey shows that 38% of youths aged 14 exclusively attended separate newcomer classes upon entering the German school system. Around 22% attended both newcomer classes and regular classes, and 40% exclusively attended regular classes upon entering the German school system (Gambaro et al., 2020). The variance appears to be large between German federal states. In North Rhine-Westphalia, administrative data for the school year 2015/2016 show that around 73.6% of refugee students in secondary education were attending newcomer classes (Emmerich et al., 2020a). Refugee children and youths were most frequently schooled separately in lower secondary schools (79.8%) and *Gymnasium* (77.3%) in North Rhine-Westphalia.

Unlike compulsory schooling for refugees and asylum seekers, there are hardly any detailed regulations for organization, configurations, duration, transitions, and quality control for newcomer classes in federal school laws (Gogolin et al., 2003; Brüggemann and Nikolai, 2016), even though a consideration of these issues in regulations and laws at the federal state and national level is now on the rise (Korntheuer and Damm, 2020). However, at the time of the school enrollment of the adolescents in our sample, there were only rough guidelines that specify, for example, whether there are any newcomer classes at all, and the maximum amount of time they should last. Even these rough guidelines vary greatly between federal states in Germany: the schooling of newly immigrated students without sufficient knowledge of German ranges from schooling in separate classes for newcomers (complete external differentiation), to a partially integrated model (partial external differentiation, i.e., when some subjects such as sport or art education are taught together), or inclusion into regular classes (internal differentiation), whereby the students often receive additional language support. For an overview of the regulations in the five federal states in our sample (see Table 2).

**Table 2:** Organization of Schooling.

**Federal state**	**Organization of schooling**
Bavaria	•Complete external differentiation; so-called “Übergangsklassen” (transitional classes) •Partial external differentiation; so-called “Deutschförderklassen” (German classes) or additional “Deutschförderkurse” (German courses) •Internal differentiation (Staatsinstitut für Schulqualität und Bildungsforschung, 2019)
Hamburg	•In secondary education, at first complete external differentiation (“Vorbereitungsklassen”–preparatory classes) in all types of school; then transition to regular classes and language support in partial external differentiation •For students aged 15 or 16, parallel model until graduation possible (Behörde für Schule und Berufsbildung, 2012)
North Rhine-Westphalia	•Complete external differentiation (no uniform notation of these separate classes) •Partial external differentiation •Internal differentiation (Ministerium für Schule und Bildung des Landes Nordrhein-Westfalen, 2018)
Rhineland- Palatinate	•Integration into regular classes with internal and partial external differentiation (Wissenschaft, Weiterbildung und Kultur, 2015; Ministerium für Bildung, 2017)
Saxony	•If language skills are insufficient: external differentiation in “Vorbereitungsklassen” (preparatory classes), then partial integration with decreasing degree of external differentiation (Sächsisches Staatsministerium für Kultus, 1992)

*Will and Homuth (2020); adapted and updated*.

Due to these large differences in legal regulations, we expect strong differences between federal states for the type of class attended. While in Rhineland-Palatinate all newly immigrated students are to be integrated into regular classes, in Hamburg there are almost exclusively separate classes for these newcomers at the time of first enrollment in Germany. In both federal states, standardization of the schooling of refugee students in a particular type of school class is high. We therefore expect that young refugees in a federal state that directly integrates newcomers into regular classes (e.g., Rhineland-Palatinate) are significantly more likely to attend a regular class than students attending school in federal states that primarily educate new immigrants separately (e.g., Hamburg). In federal states with more de-standardization, which do not rule out integrated schooling (e.g., Bavaria, North Rhine-Westphalia, and Saxony), we expect lower probabilities of attending a regular class than in federal states that educate newcomers directly in regular classes, but the differences should not be as pronounced as to federal states that only educate newcomers separately. We further expect that individual resources, especially the education of the parents and the previous educational experience of the adolescents, have an additional influence. While parents have limited ability to influence the assignment process itself, parents' education and students' prior schooling experiences should have an impact on individuals' performance in school, thereby influencing the kind of class to which they are assigned^5^.

#### Type of School Attended

Previous findings suggest that refugee students in Germany are more likely to attend lower secondary schools than *Gymnasium* (for an overview, see Winkler, 2021). According to representative data for all German federal states, 33.5% of refugee students in grade nine attended lower secondary schools, while only 8.1% went to *Gymnasium* (Henschel et al., 2019). Administrative data for the German secondary school system also show that students with Syrian citizenship are less likely to attend a *Gymnasium*. In the school year 2014/15, the attendance rate for Syrians at *Gymnasium* in Hamburg was 15.7%, followed by Saxony (11.9%), Rhineland-Palatinate (11.1%), and North Rhine-Westphalia (11.1%). In Bavaria, only 4.7% of Syrian students attend a *Gymnasium* (El-Mafaalani and Kemper, 2017)^6^.

Similar to the organization and curricula of newcomer classes, the placement into a type of school at which newly immigrated students are enrolled is not regulated uniformly throughout Germany. Table 3 provides an overview of the regulations in the five federal states in our sample. Whereas, in some states (Bavaria, Saxony), newcomer classes are mainly offered at certain, usually less demanding, types of schools, in other states there are newcomer classes in all types of schools (Hamburg, North Rhine-Westphalia). In Rhineland-Palatinate, where newly arrived immigrants should be integrated into regular classes directly, parents and students can choose between all school types.

**Table 3:** Assignment to type of school and grade level.

**Federal state**	**Assignment to a type of school and grade level**
Bavaria	Initially, refugees are mainly enrolled in lower secondary schools (*Mittelschule*). If students show a particular aptitude, a subsequent schooling at intermediate and higher secondary school tracks is possible–initially as a guest student. In general, the transition to a regular class should be completed after 2 years (Staatsinstitut für Schulqualität und Bildungsforschung, 2019).
Hamburg	Assignment to a school track occurs under consideration of educational experiences prior to enrollment in age-appropriate preparatory classes (all types of school possible). Transition to a regular class depends on the students' age. Retention in preparation classes should not exceed 1 year (Behörde für Schule und Berufsbildung, 2012).
North Rhine-Westphalia	Assignment to a regular class at a specific school track according to individual achievement, individual learning development, and expected performance as soon as German language skills are sufficient (if possible, at the latest after 2 years); all type of schools possible (Ministerium für Schule und Bildung des Landes Nordrhein-Westfalen, 2018).
Rhineland-Palatinate	Choice of type of secondary school is the responsibility of parents or adult students (all type of schools possible) (Ministerium für Bildung, 2017). Grade level should correspond to the age and previous educational experiences of students (Wissenschaft, Weiterbildung und Kultur, 2015).
Saxony	Preparatory classes are mainly at *Oberschule* (combined lower and intermediate track). The duration of attendance of preparatory classes is usually 1 year. As soon as students have sufficient German language skills, they transition to regular classes, usually at the beginning of a school semester, according to their age or level of performance (Sächsisches Staatsministerium für Kultus, 1992).

*Will and Homuth (2020); adapted and updated*.

Between federal states, we expect differences in educational participation in different types of schools (see Table 3). Since newcomer classes are not offered at *Gymnasium* in Bavaria and Saxony, we expect strong path dependencies from this standardized educational pathway: refugees are presumably less likely to attend *Gymnasium* in these federal states, even if they have moved from a newcomer class to a regular class in the meantime. In Hamburg and North Rhine-Westphalia, where newcomer classes are also offered at *Gymnasium*, and in Rhineland-Palatinate, where newcomers are enrolled in regular classes of all school types, the proportion of refugees on the academic track should be greater. We also expect that parental education and the previous school performance of the young refugees are positively correlated with attending a *Gymnasium*.

#### Schooling in an Age-Appropriate Grade Level

Regarding the last of our educational domains, we analyze whether students are enrolled in a suitable grade level for their age. Empirical findings on age-appropriate placement into school grades of refugee students is scarce. One study has shown that the share of refugee students aged 11–14 years who are still attending primary education is 20%, while the share of non-immigrant students of the same age in primary education is only 11% (de Paiva Lareiro, 2019).

The criteria used to assign newly immigrated students to a specific grade level vary from federal state to federal state, with age and prior achievement mentioned in the regulations relatively often. We suspect, however, that it is more relevant *when* the young people are assigned to a specific grade level. We assume that federal states which assign refugee students directly to a certain school track and a certain grade level (according to Table 3 Hamburg and Rhineland-Palatinate) might use age as a central criterion for assignment to a class level. In these cases, for school administrations, age may serve as a proxy for the assumed cognitive development and accumulated knowledge of the students. In contrast, federal states which first integrate refugee students into newcomer classes without direct assignment to a school track and a grade level might have more time to evaluate the performance of the students. These federal states might be more cautious and aim to avoid placing a student into a too demanding learning environment. Thus, these federal states might assign students not only by their age, but may also consider, for example, their skill level or their social competencies. We would therefore expect those federal states which assign refugee students directly to a certain school track and a certain grade level to assign students to a grade that is more suitable for their age. In federal states where the assignment to a grade level takes place later (e.g., Bavaria, Saxony, and North Rhine-Westphalia), we assume that also other aspects, such as performance or behavior play a more central role. In sum, variation between federal states in the age-appropriate assignment of students occurs due to more extensive regulations in some federal states, which allows them to deviate from age norms. Variation is, however, not likely to arise from more possibilities for individual decision-making: we assume that the students' parents cannot simply override their offsprings' school assignment. Therefore, refugee families should have relatively little direct influence on the assignment process. However, we expect that family resources (e.g., the educational background of the parents) or individual resources (previous educational experiences) positively correlate with age-appropriate schooling, as they directly affect students' school achievement and therefore the assignment to a grade level by the school administration.

## Data and Methods

### Data

Our analyses are based on the first wave of the data collected within the context of the “Refugees in the German Educational System (ReGES)” study, which was conducted in spring 2018 (Will et al., 2021)^7^. The study includes refugee children and adolescents who migrated to Germany since 2014, are living in Germany with a least one parent or legal guardian^8^, and have already been allocated to a municipality. We understand refugees as persons seeking humanitarian protection in Germany, who have applied, or intend to apply for asylum in Germany. The sample was drawn from the municipality resident registration offices (for more details, see Steinhauer et al., 2019). Due to the sampling procedure, which overrepresents nationals of the ten most common origin countries of refugees in 2015 and 2016 in Germany with high prospects of being granted asylum, refugee adolescents with secure resident status are overrepresented. The data were obtained in five German federal states, which vary according to selected macro factors. Besides the number of refugees admitted^9^, another central reason was the fact that the federal states differ, particularly regarding their schooling strategies for newly arrived underage immigrants (e.g., Steinhauer et al., 2019). We focus on the adolescent cohort (Refugee Cohort 2), which includes 2,415 young adolescents aged 14–16, who were still in lower secondary education at the first measurement point^10^. The high proportion of Syrian refugees in Germany is also reflected in our data, which consists of 69% Syrian students. All information used in our analyses, including data on the school situation, are collected through surveys of refugee adolescents and their parents (for a more detailed description of research design, sampling, and response rates, see also Will et al., 2021).

### Operationalization and Descriptive Statistics

#### Dependent Variables

The dependent variables are operationalized as follows. The wait time up to school enrollment in Germany is measured in months and calculated as the month and year of first enrollment in Germany minus the month and year of arrival in Germany. Plausibility checks were applied that consider that the time of enrollment must not precede the time of arrival. The type of class attended includes information on whether a student has ever attended a newcomer class (yes vs. no). The type of school attended is measured as *Gymnasium* vs. other school types. Graduating from *Gymnasium* allows students to pursue higher education, while other school types tend to channel students mainly into vocational training. We therefore chose an operationalization that attributes comprehensive schools to other school types, even though these schools might also comprise academic tracks. In many instances, refugee students were placed into a newcomer class regardless of the school type because the respective school had positions in a newcomer class available. When refugee students transfer to a regular class, they sometimes change school and school type (Emmerich et al., 2020b). To avoid biases in this regard, only students who already attend regular classes are considered. Age-appropriate placement is assessed with a variable that indicates whether the students' age corresponds to the regular age of students attending the same grade level. The variable measures whether the current age (in years) minus seven years (6 years as average enrollment age in Germany plus 1 year leeway) equals the grade level. That means that a person who is, for example, 16 years old and attends at least grade 9 is coded age-appropriately placed. Grade levels that were not plausible due to the sampling procedure (below grade 5) were excluded from the analysis (see Table 4 for an overview of all dependent variables, see Appendix A for an overview of all variables by federal state).

**Table 4:** Overview of dependent and control variables.

	**N**	**Missing values**	**%/M**	**SD**	**Min**	**Max**
**Dependent variables**						
Duration up to school enrollment (in months)	2,214	201	7.20	6.74	0	51
Type of class attended	2,410	5	0.50	0.50	0 (regular)	1 (newcomer)
Type of school attended^a^	1,530	3	0.19	0.39	0 (other)	1 (*Gymnasium*)
Age-appropriate placement	2,396	19	0.68	0.47	0 (not age-appropriate)	1 (age-appropriate)
**Social origin**						
Highest parental ISCED	2,017	398				
No/less than primary education			34.56%			
Primary education			9.82%			
Secondary I + II education			30.54%			
Postsecondary/tertiary education			25.09%			
Highest ISEI-08 (parents)	1,912	503	44.49	25.84	0	88.96
**Previous educational experiences**						
Self-assessed school performance	2,164	251	76.19	20.80	0	100
**Control variables**						
Urbanity	2,415	0				
>500.000 inhabitants			58.39%			
100.000–500.000 inhabitants			29.07%			
<100.000 inhabitants			12.55%			
Sex	2,415	0				
Male			55.07%			
Female			44.93%			
Age (at arrival, in months)	2,415	0	161.95	13.73	124	205
Origin country	2,415	0				
Afghanistan			9.03%			
Iraq			13.17%			
Syria			68.82%			
Other			8.99%			
Resident status	2,102	313				
Secure			68.84%			
Insecure			31.16%			
Current type of class	2,412	3				
Regular class			63.56%			
Newcomer class			36.44%			

*^a^Only students who attend a regular class are included in the calculation*.

*ReGES data, Refugee Cohort 2–Adolescents, doi: 10.5157/ReGES:RC2:SUF:2.0.0.*

*The descriptive statistics for the independent variables are based on the overall sample before imputation and slightly deviate across each set of analyses due to varying number of cases*.

#### Independent and Control Variables

##### Legal Regulations

The main independent variable is the difference in regulations between federal states. To analyze *differences in regulations*, variables are generated that group the federal states according to their regulations for each of the dependent variables. Thus, the variable measuring differences in legal regulations differs across analyses. Table 5 provides an overview of the regulations and the respective variable for each set of analyses.

**Table 5:** Overview of categorization of legal regulations per dependent variable.

	**Categories**	**Federal States**	**Distribution**
Duration up to enrollment	1. Without delay 2. Three months after moving from abroad 3. After assignment to municipality	•Hamburg •Bavaria •North Rhine-Westphalia, Rhineland-Palatinate, Saxony	10.70% 12.15% 77.15%
Type of class	1. External + partially external differentiation 2. External, partially external and internal differentiation 3. Partially external + internal differentiation	•Hamburg •Bavaria, North Rhine-Westphalia, Saxony •Rhineland-Palatinate	11.66% 76.43% 11.91%
Type of school	1. Enrollment mainly at lower school types 2. Enrollment more flexible	•Bavaria, Saxony •Hamburg, North Rhine-Westphalia, Rhineland-Palatinate	18.91% 81.09%
Age-appropriate placement	1. Assignment to a grade level upon enrollment 2. Assignment to a grade level later	•Hamburg, Rhineland-Palatinate •Bavaria, North Rhine-Westphalia, Saxony	23.54% 76.46%

*ReGES data, Refugee Cohort 2–Adolescents, doi: 10.5157/ReGES:RC2:SUF:2.0.0*.

*Values are based on the original data. Cases for which the respective dependent variable was missing are not considered*.

The further independent and control variables encompass different factors that are assumed to further influence adolescents' educational participation. Besides differences in regulations, we include variables measuring social origin, previous educational experiences, and a variety of macro- and micro-level control variables.

##### Social Origin

For social origin we use two measures. For parental educational background, we use highest parental educational level measured by the International Standard Classification of Education (ISCED-1997). The ISCED-index is based on the CAMCES (Computer-Assisted Measurement and Coding of Education in Surveys) instrument, with alternative coding (see Schneider et al., 2018). The CAMCES instrument allows qualifications obtained in different countries to be coded according to the ISCED-scheme. Additionally, cases with no education are grouped with cases with less than primary education. Categories were summarized to achieve sufficient numbers per category (see Table 4 for an overview). The categories encompass “no/less than primary education,” “primary education,” “secondary I + II education,” “postsecondary/tertiary education.” Parental socioeconomic background is measured using the highest parental occupational status in the origin country as measured according to the International Socio-Economic Index of Occupational Status (ISEI). The scale ranges from 11.01 to 88.96. Additionally, if no parent was employed in the country of origin, the highest ISEI was coded as 0.

##### Previous Educational Experiences

Previous educational experiences are measured using self-assessed school performance in the origin country (scale from 0 to 100). The alternative measure “years of schooling in the origin country” is not considered as it correlates too strongly with the adolescents' ages.

##### Control Variables on the Macro- and Micro-Level

Control variables include structural factors such as the degree of urbanity and the timing of arrival in Germany, which might influence the allocation to and availability of schools. The degree of urbanity is divided into three categories “more than 500.000 inhabitants,” “100.000–500.000 inhabitants,” and “ <100.000 inhabitants.” The timing of arrival includes the number of adolescents in our sample who arrived in a certain quarter (ranging from 01/2014 to 04/2017, with a peak in 04/2015). This variable was only controlled for in the first set of analyses (duration up to school enrollment). Further control variables on the micro-level include sex, age, country of origin, and resident status. Sex was coded as female or male. Age was measured as age upon arrival (in months). The country of origin includes the main origin countries: Afghanistan, Iraq, and Syria. Countries of origin with <3% cases were subsumed under the category “other.” The current resident status was coded as secure vs. unsecure. We coded the resident status as secure if they are recognized as a refugee, their application for asylum has been accepted, or if they have been given a different protection status. Insecure resident status entails those whose application was denied but were allowed to stay short-term in Germany, those whose application was denied and were asked to leave Germany, those who had not received a decision on their application, as well as those who had not yet submitted an application. The high percentage of adolescents with secure status is due to the sampling strategy, which overrepresents refugees from nationalities with good prospects of staying in Germany (e.g., Steinhauer et al., 2019). Whether a refugee student attended a newcomer class at the time of the survey is measured as yes vs. no (only controlled for in the analyses on age-appropriate placement).

### Analytical Strategy

Our analytical strategy is based on stepwise robust linear regressions (OLS), and stepwise linear probability models (LPM) with robust standard errors. Analyses are carried out using Stata. We use iterated chained equations (White et al., 2011) to multiply impute missing data for all independent variables (see Table 4). We also imputed missing values for the outcome variable, but deleted them after the imputation (see von Hippel, 2007, 2020). Following von Hippel (2020), we applied a quadratic rule to determine the required number of imputations (M = 73) based on the fraction of missing information in our fully specified model^11^.

For each dependent variable, we applied a stepwise procedure. We first calculated a model that only considers the main independent variable (i.e., legal regulations). Afterwards, we introduced the variables measuring social origin, then self-assessed school performance in the origin country. The last model additionally includes the control variables at the macro and micro level (see Independent and Control Variables). We run a stepwise OLS regression on the duration until school enrollment, and stepwise linear probability models on attending a newcomer class, attending *Gymnasium*, and age-appropriate placement. There are only slight differences in the models. First, we generated a different variable containing legal regulations for each dependent variable (see Table 5). Second, we additionally controlled for the time of arrival in our analyses on the duration until school enrollment. Third, we controlled for whether the students attended a refugee class at the time of the survey in our analyses on age-appropriate placement.

## Results

### Duration Until School Enrollment in Germany

**Figure 1:**
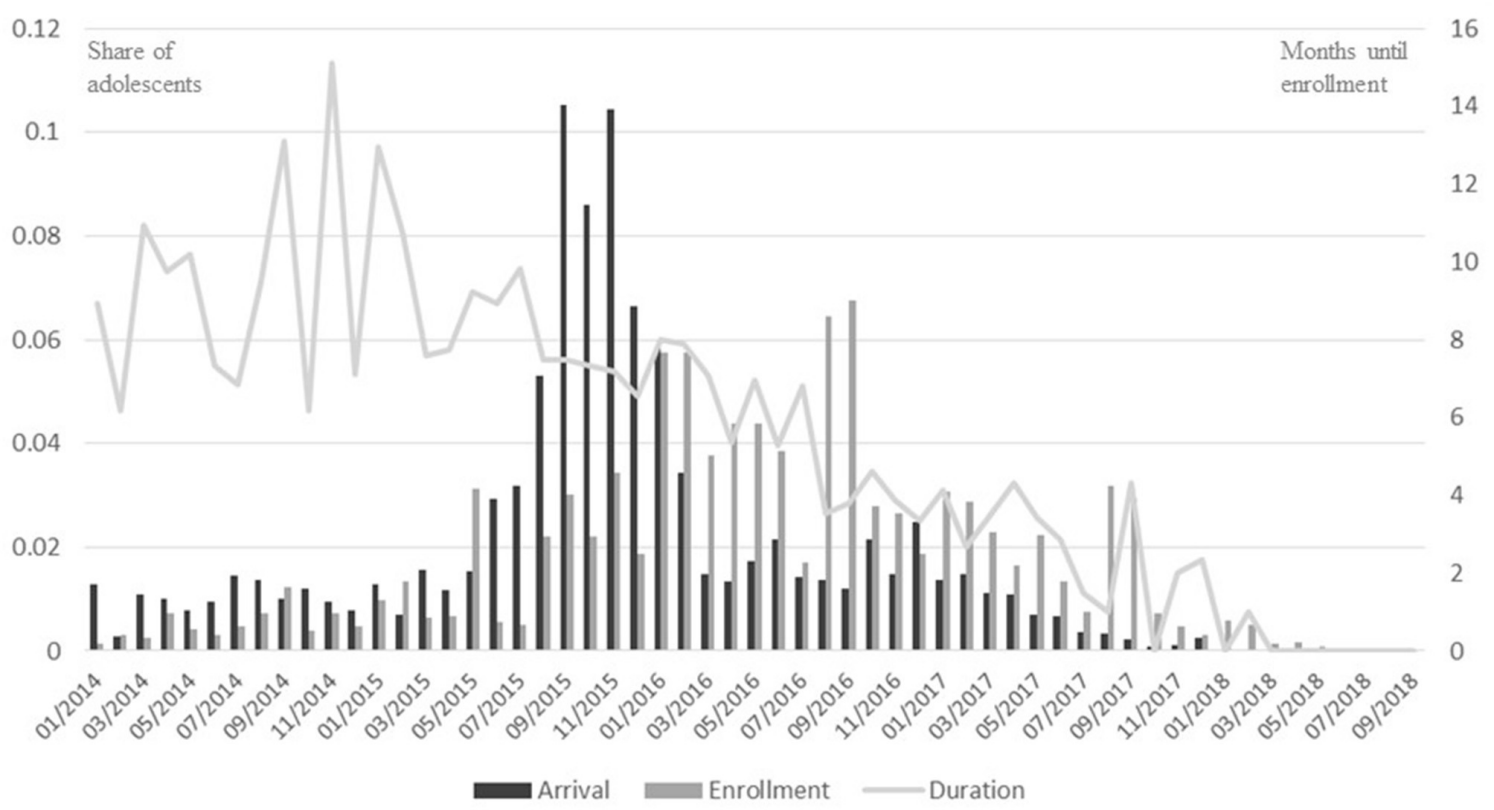
Arrival time, enrollment time, and enrollment duration of refugee adolescents by month (2014–2018). ReGES data, Refugee Cohort 2–Adolescents, doi: 10.5157/ReGES:RC2:SUF:2.0.0. “Arrival” and “Enrollment” indicate the share of the adolescents surveyed who arrived (*n* = 2,415) and enrolled (*n* = 2,367) during a given month (left axis). “Duration” indicates the average duration until enrollment from the time of arrival (right axis, *n* = 2,214). The figure is based on the original data.

The descriptive analysis shows that the adolescents surveyed are, on average, enrolled at school about seven months after arriving in Germany (see also Homuth et al., 2020), with large differences ranging from direct enrollment after arriving, up to 51 months' wait time (standard deviation of 6.74 months, see Table 4). The duration seems to depend on the time point within the school year at which the adolescents arrived. An arrival time closer to the beginning of the school year (for example in early summer) and the half year (e.g., in January) generally led to a quicker enrollment. Additionally, students who arrived later seem to be enrolled faster, probably because numbers of arriving refugee students were lower, and structures to school refugee children had already been implemented (see Figure 1).

Table 6 displays the results of the stepwise OLS regressions. As a reminder, the main independent variable (i.e., legal regulations) measures whether refugee students are enrolled without delay after entering the federal state (i.e., Hamburg), 3 months after moving to Germany (i.e., Bavaria), or whether they are enrolled after being assigned to a municipality (i.e., North Rhine-Westphalia, Rhineland-Palatinate, Saxony; see also Table 5). We used the largest category (i.e., after being assigned to a municipality) as the reference category. The results show that students in federal states in which compulsory education starts without delay, or 3 months after moving from abroad, have a shorter waiting period until enrollment compared to the students in federal states in which compulsory education starts only after students have been assigned to a municipality (Model 1.1). The values of −1.855 (compulsory enrollment 3 months after moving) and −0.156 (compulsory schooling without delay) indicate that students in these federal states are enrolled almost two months respectively almost less than a week earlier than students in federal states in which compulsory education starts after assignment to a municipality. However, in this basic model, this relationship is only significant for students in federal states in which compulsory schooling starts 3 months after moving from abroad.

**Table 6:** Stepwise OLS regression on duration until school enrollment as dependent variable.

	**Model 1.1**	**Model 1.2**	**Model 1.3**	**Model 1.4**
**Legal regulations: enrollment…**				
(*ref.: …after assignment to municipality)				
…without delay	−0.156	−0.334	−0.388	−1.169*
	(0.456)	(0.465)	(0.466)	(0.500)
…three months after moving from abroad	−1.855***	−1.914***	−1.993***	−2.282***
	(0.398)	(0.397)	(0.398)	(0.384)
**Highest parental ISCED**				
(*ref.: secondary I+II education)				
No/less than primary education		0.932*	0.793*	0.557
		(0.398)	(0.399)	(0.402)
Primary education		1.313*	1.268*	1.352*
		(0.645)	(0.644)	(0.619)
Postsecondary/tertiary education		0.078	0.125	0.146
		(0.467)	(0.468)	(0.449)
**Highest ISEI-08 (parents)**		−0.013	−0.011	−0.011
		(0.008)	(0.008)	(0.008)
**Average school performance (origin country)**			−0.019*	−0.021**
			(0.007)	(0.007)
**Degree of urbanity**				
(*ref.: >500.000 inhabitants)				
100.000–500.000 inhabitants				−0.333
				(0.323)
<100.000 inhabitants				−0.482
				(0.478)
**Gender**				
Female				0.260
				(0.282)
**Age at arrival (in months)**				0.018
				(0.014)
**Origin country**				
(*ref.: Syria)				
Afghanistan				0.710
				(0.518)
Iraq				0.393
				(0.407)
Other				−0.125
				(0.509)
**Resident status**				
Insecure resident status				−1.369***
				(0.301)
R^2^	0.0080	0.0193	0.0225	0.1054

*ReGES data, Refugee Cohort 2–Adolescents, doi: 10.5157/ReGES:RC2:SUF:2.0.0*.

The time of arrival (in quarters) has been controlled for but is omitted from the table (see Appendix B for the full model). The results present the regression coefficients and their standard errors in parentheses. Significance levels are marked as follows: ^***^p < 0.001, ^**^ p < 0.01, ^*^ p < 0.5. n = 2,214. Imputed data, M = 73.

When controlling for social origin (Model 1.2), the effects of legal regulations slightly increase. Additionally, we observe–as might be expected–that social origin has, at least partially, an effect on the time until enrollment. Students with parents who have obtained only primary-level education or less (compared to students with parents who have obtained secondary-level education) are more likely to be enrolled a little bit later. The negative effect for self-assessed school performance (Model 1.3), indicating a quicker enrollment for better students is significant, but seems very small (−0.019). However, self-assessed school performance is measured on a scale from 0 to 100, which means that adolescents who consider their performance in the country of origin to be very good (scale value = 100) are enrolled at school almost 1 month earlier than adolescents who consider their performance to be more middling (scale value = 50). When controlling for further variables on the macro and micro level (Model 1.4), we observe that the effects of legal regulations are both significant and even stronger than in the basic model. This confirms our assumption that in federal states in which compulsory education starts only after being assigned to a municipality, refugee adolescents have to wait longer to be enrolled. The multivariate analyses roughly confirm the above-mentioned connection between time of arrival (see Figure 1; for the individual regression coefficients of the arrival quarters see Appendix B), showing that students arriving between mid-2016 and 2017 are indeed significantly more likely to be enrolled faster. It can be assumed that the faster enrollment is due the lower number of arriving refugee students, as well as the fact that administrative procedures and structures to school refugee children became more established over time. Changes in regulations, which might also explain a faster enrollment, are not observed during the period under study. Other factors such as the degree of urbanity, sex, age, and origin country seem to have no significant effect on the duration until enrollment. The effect of self-assessed school performance in the origin country remains significant and even becomes a little bit stronger than in the previous models. Resident status seems to correlate with wait times, with surprisingly, students with insecure resident status being enrolled quicker.

Overall, it can be stated that the relationship between legal regulations and duration until enrollment is very strong and robust across models. In the full model (Model 1.4), the effects of legal regulations are most pronounced and statistically significant showing slower enrollment in federal states in which schooling starts after assignment to a municipality. Thus, delaying the start of compulsory schooling until families are assigned to a municipality has the consequence of further increasing youths' interruption of schooling for, on average, another one to two months.

### Type of Class Attended

The descriptive analysis shows that about half of the students have attended a newcomer class during their school education in Germany (see Table 4).

Table 7 shows the results of the stepwise linear probability models. As a reminder, the variable on legal regulations contains three categories, direct integration into regular classes (i.e., Rhineland-Palatinate), primarily enrollment in separate classes (i.e., Hamburg), and more flexible assignment to either a regular or newcomer class (i.e., Bavaria, North Rhine-Westphalia, and Saxony, see also Table 5). We used the largest category (i.e., more flexible assignment) as a reference category. In line with our assumptions, the results indicate that students in federal states who integrate newly immigrated students directly into regular classes are indeed less likely to attend a refugee class (compared to those with a more flexible assignment to either a regular or newcomer class, Model 2.1). The value of −0.314 indicates that adolescents attending school in federal states which directly integrate newcomers in regular classes are, with a probability of 31.4 percentage points, indeed less likely to attend a newcomer class. Students in federal states which almost exclusively assign students to newcomer classes at first are, with a probability of 6.5 percentage points, more likely–as assumed–to attend a newcomer class (in comparison to the reference group). These effects remain stable if we control for social origin (Model 2.2), self-assessed school performance in the origin country (Model 2.3), and further control variables on the macro and micro level (Model 2.4), with the effect for federal states with direct integration into a regular class slightly decreasing, and the effect for federal states which almost exclusively assign students to newcomer classes at first increasing slightly.

**Table 7:** Stepwise linear probability models on attending a newcomer class as dependent variable (regular class vs. newcomer class).

	**Model 2.1**	**Model 2.2**	**Model 2.3**	**Model 2.4**
**Legal regulations**				
(*ref.: more flexible assignment)				
External differentiation	0.065*	0.070*	0.070*	0.087*
	(0.031)	(0.032)	(0.032)	(0.034)
Internal differentiation	−0.314***	−0.311***	−0.310***	−0.266***
	(0.027)	(0.027)	(0.027)	(0.031)
**Highest parental ISCED**				
(*ref.: secondary I+II education)				
No/less than primary education		−0.066*	−0.065*	−0.042
		(0.027)	(0.028)	(0.028)
Primary education		−0.018	−0.018	−0.020
		(0.041)	(0.041)	(0.041)
Postsecondary/tertiary education		−0.070*	−0.070*	−0.071*
		(0.033)	(0.033)	(0.032)
**Highest ISEI-08 (parents)**		−0.001	−0.001	−0.001
		(0.001)	(0.001)	(0.001)
**Average school performance (origin country)**			0.000	0.000
			(0.001)	(0.001)
**Degree of urbanity**				
(*ref.: >500.000 inhabitants)				
100.000–500.000 inhabitants				−0.014
				(0.024)
<100.000 inhabitants				−0.110**
				(0.034)
**Gender**				
Female				−0.033
				(0.020)
**Age at arrival (in months)**				0.003***
				(0.001)
**Origin country**				
(*ref.: Syria)				
Afghanistan				−0.125**
				(0.038)
Iraq				−0.016
				(0.031)
Other				−0.046
				(0.035)
**Resident status**				
Insecure resident status				0.034
				(0.023)
R^2^	0.0453	0.0524	0.0525	0.0718

*ReGES data, Refugee Cohort 2–Adolescents, doi: 10.5157/ReGES:RC2:SUF:2.0.0*.

The results present the regression coefficients and their standard errors in parentheses. Significance levels are marked as follows: ^***^p < 0.001, ^**^p < 0.01, ^*^ p < 0.5. n = 2,410. Imputed data, M = 73.

Our assumption that children from families with a high educational background were more likely to attend a regular class cannot be satisfactorily confirmed, as the results are ambiguous. While we observe a small negative effect (i.e., being less likely to attend a newcomer class) for children whose parents have a postsecondary or tertiary education, we also see a negative effect of a similar magnitude for adolescents whose parents have obtained no or less than primary education (compared to adolescents whose parents obtained secondary-level education) (Model 2.2). This effect for adolescents whose parents have obtained no or less than primary education, however, is insignificant when controlling for further variables on the macro and micro level (Model 2.4). Contrary to our expectation, the adolescents' self-assessed school performance in their origin country is not significantly related to attending a regular class. Thus, our assumption that families' educational background, and adolescents' previous educational achievements are relevant for their assignment to a certain type of class are not confirmed by our analyses.

Overall, it appears that family and individual resources are not relevant for the type of class in which refugee students are enrolled. The decisive factor seems to be the legal regulations, which–depending on the assigned federal state–promote or reduce separate schooling^12^.

### Access to Different Types of Schools

Almost 21.90% of the adolescents surveyed attended a *Gymnasium*, while the remaining 78.10% attended either a *Hauptschule*, a *Realschule*, a comprehensive school or a joint school. Considering only students who are already assigned to a regular class, the share of students at a *Gymnasium* is even lower (18.69%). This distribution is in line with previous findings that refugee students in Germany are more likely to attend lower secondary schools than a *Gymnasium*.

Table 8 displays the results from the stepwise linear probability models with the type of school attended as dependent variable. The legal regulations suggest that refugee students who attend school in federal states in which newly migrated students are assigned to newcomer classes only at lower school types (i.e., Bavaria, Saxony), are less likely to attend a *Gymnasium* than refugee students in federal states that are more flexible in assigning students to different school types (i.e., Hamburg, North Rhine-Westphalia, Rhineland-Palatinate). Our analysis confirms our hypothesis: refugee students in federal states which tend to assign newly migrated students to lower school types are indeed, with a probability of around 16 percentage points, less likely to attend a *Gymnasium* than students in federal states that are more flexible in assigning students to different school types (Model 3.1). This finding is all the more remarkable because we only consider students who are already attending a regular class, and who can therefore be assumed to have already been definitively assigned to a type of school. These effects remain stable even if we control for social origin (Model 3.2), self-assessed school performance (Model 3.3), and further control variables (Model 3.4). Additionally, we find that students with parents who obtained postsecondary or tertiary level education are significantly more likely to attend a *Gymnasium* (than students with parents who obtained secondary education, Model 3.2–3.4). Furthermore, the self-assessed school performance in the country of origin (and also the adolescents' age) has a significant, though rather small positive effect on the likelihood of attending a *Gymnasium*. That means that older students and students who assess their school performance as better are slightly more likely to attend a *Gymnasium*. These findings are in line with our previous assumptions on the relationship between parental education and previous school performance on *Gymnasium* attendance^13^.

**Table 8:** Stepwise linear probability models on attending *Gymnasium* as dependent variable (other school vs. *Gymnasium*).

	**Model 3.1**	**Model 3.2**	**Model 3.3**	**Model 3.4**
**Legal regulations**				
(*ref.: enrollment more flexible)				
Enrollment at lower school types	−0.161***	−0.158***	−0.153***	−0.161***
	(0.018)	(0.018)	(0.018)	(0.019)
**Highest parental ISCED**				
(*ref.: secondary I+II education)				
No/less than primary education		−0.022	−0.005	−0.013
		(0.027)	(0.027)	(0.028)
Primary education		−0.020	−0.017	−0.024
		(0.039)	(0.039)	(0.040)
Postsecondary/tertiary education		0.101**	0.097**	0.094**
		(0.034)	(0.033)	(0.034)
**Highest ISEI-08 (parents)**		0.000	0.000	0.000
		(0.001)	(0.001)	(0.001)
**Average school performance (origin country)**			0.002***	0.002***
			(0.001)	(0.001)
**Degree of urbanity**				
(*ref.: >500.000 inhabitants)				
100.000–500.000 inhabitants				−0.023
				(0.022)
<100.000 inhabitants				−0.051
				(0.029)
**Gender**				
Female				0.029
				(0.020)
**Age at arrival (in months)**				0.002***
				(0.001)
**Origin country**				
(*ref.: Syria)				
Afghanistan				0.017
				(0.035)
Iraq				0.001
				(0.031)
Other				−0.019
				(0.034)
**Resident status**				
Insecure resident status				−0.019
				(0.023)
R^2^	0.0269	0.0501	0.0647	0.0756

*ReGES data, Refugee Cohort 2–Adolescents, doi: 10.5157/ReGES:RC2:SUF:2.0.0*.

The results present the regression coefficients and their standard errors in parentheses. Significance levels are marked as follows: ^***^p < 0.001, ^**^p < 0.01, ^*^ p < 0.5. n = 1,530. Imputed data, M = 73.

To sum up, this means that the legal regulations concerning assignment to a school type, which differ between federal states, constitute a decisive factor that determines the starting conditions for students' further educational pathways, and strongly pre-structures their educational trajectories^14^. However, we also see a stronger influence of family and individual resources here, so that there seems to be at least partial scope for decision-making among refugee families when it comes to choosing a type of school.

### Age-Appropriate Placement in a School Class

**Figure 2:**
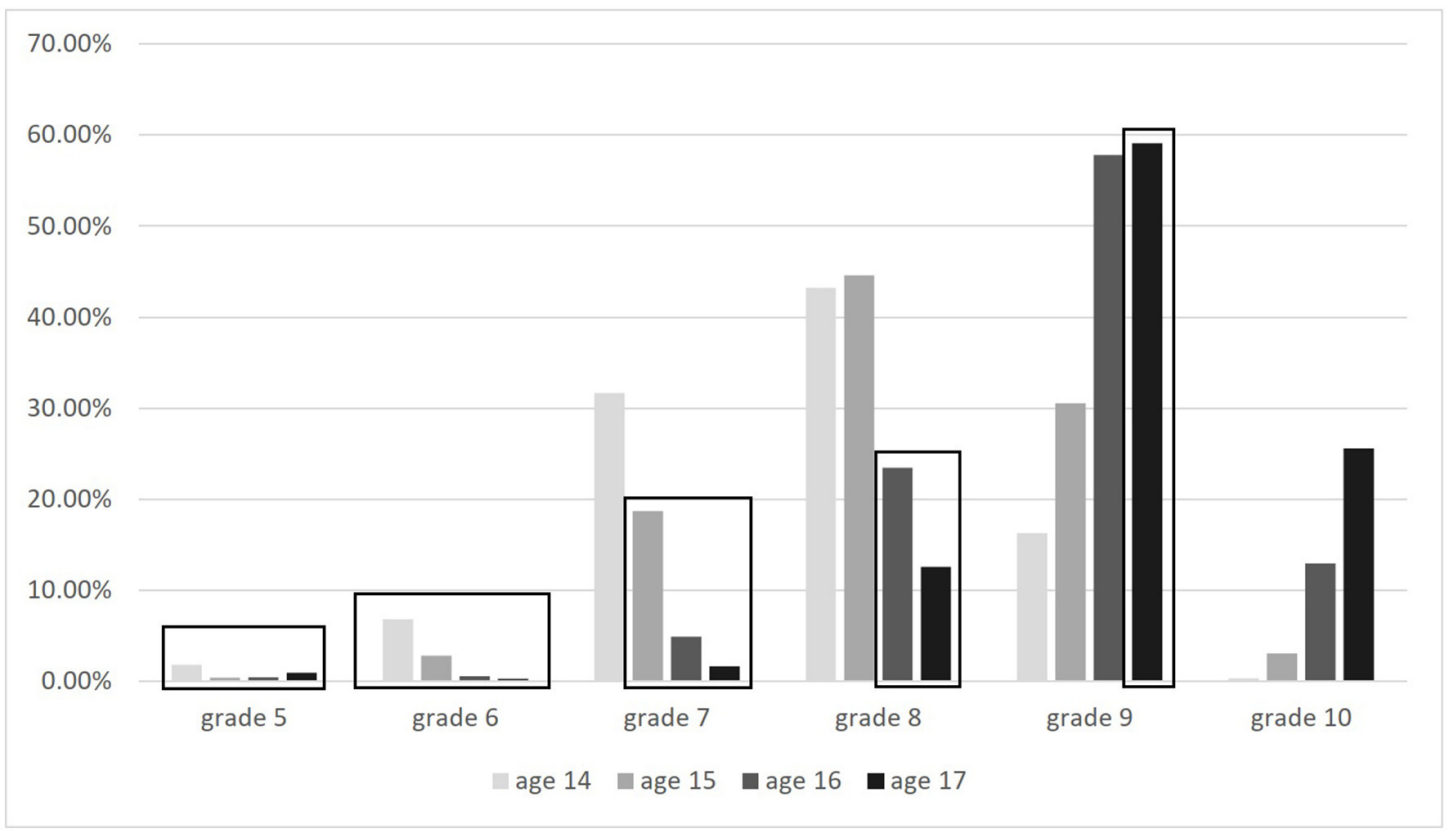
Age and school grade of the refugee adolescents. ReGES data, Refugee Cohort 2–Adolescents, doi: 10.5157/ReGES:RC2:SUF:2.0.0. Highlighted in black boxes are cases which are not age-appropriately enrolled by our definition *n* = 2,396.

The age of the adolescents surveyed ranges between 14 and 17 at the time of interview, with the majority being either 15 (35.78%) or 16 years (31.80%) old. Most of them either attend grade nine (42.40%) or grade eight (31.84%). A first look at the distribution of adolescents' ages and the attended grade levels (Figure 2) shows that the majority of those aged 14, as well as those aged 15, attend grade eight, whilst the largest share of adolescents who are 16 or 17 years old attend grade nine. Recalling our operationalization of age-appropriate enrollment, adolescents who are 17 years old should at least attend grade ten, adolescents who are 16 years old should at least attend grade nine and so on. The descriptive analyses show that the majority of the refugee adolescents attends at least the grade level that is appropriate for their age (overall, 68.07%). Conversely, however, this also means that almost a third of the adolescents in our sample attend a class that does not correspond to their age. Particularly older students seem to attend lower grade levels.

Table 9 displays the results from our stepwise linear probability models on age-appropriate placement as a dependent variable. As a reminder, the variable on legal regulations measures whether refugee students are assigned to a school track and grade level upon enrollment (i.e., Hamburg, Rhineland-Palatinate), and whose assignment is thus assumed to be more strongly connected to their age compared to students in federal states which do not directly assign students to a school track and grade level (i.e., Bavaria, Saxony, and North Rhine-Westphalia). Our analyses show that students in federal states which assign students directly to a grade level and school track are indeed more likely to be age-appropriately placed (see Table 9). This significant effect even slightly increases when controlling for further variables (Model 4.2–4.4). We expected that family resources (e.g., educational background of the parents), or individual resources (previous educational achievement) positively correlate with age-appropriate schooling. Initially, this does not seem to be the case (Model 4.2 and 4.3). Only in the full model (Model 4.4) are students with parents who obtained no or less than primary education (compared to those who obtained secondary education) slightly less likely to be age-appropriately placed. Overall however, the opportunities for students and their parents to exert influence here appears to be relatively small. Furthermore, as our descriptive analyses suggested, older students are slightly less likely to be the same age as their classmates, but are indeed older than the average student.

**Table 9:** Stepwise linear probability models on age-appropriate placement as dependent variable (not age-appropriate placement vs. age-appropriate placement).

	**Model 4.1**	**Model 4.2**	**Model 4.3**	**Model 4.4**
**Legal regulations**				
(*ref.: assignment to grade level later)				
Assignment to grade level upon enrollment	0.107***	0.111***	0.112***	0.125***
	(0.021)	(0.021)	(0.021)	(0.022)
**Highest parental ISCED**				
(*ref.: secondary I+II education)				
No/less than primary education		−0.043	−0.041	−0.055*
		(0.026)	(0.027)	(0.027)
Primary education		−0.043	−0.043	−0.047
		(0.039)	(0.039)	(0.038)
Postsecondary/tertiary education		0.030	0.029	0.031
		(0.030)	(0.030)	(0.030)
**Highest ISEI-08 (parents)**		−0.000	−0.000	0.000
		(0.001)	(0.001)	(0.001)
**Average school performance (origin country)**			0.000	0.000
			(0.000)	(0.000)
**Degree of urbanity**				
(*ref.: >500.000 inhabitants)				
100.000–500.000 inhabitants				−0.016
				(0.021)
<100.000 inhabitants				−0.015
				(0.029)
**Gender**				
Female				0.035
				(0.018)
**Age at arrival (in months)**				−0.010***
				(0.001)
**Origin country**				
(*ref.: Syria)				
Afghanistan				0.053
				(0.032)
Iraq				0.063*
				(0.028)
Other				0.025
				(0.032)
**Resident status**				
Insecure resident status				0.028
				(0.022)
**Refugee class**				
Currently attending refugee class				0.019
				(0.019)
R^2^	0.0095	0.0140	0.0143	0.1074

*ReGES data, Refugee Cohort 2–Adolescents, doi: 10.5157/ReGES:RC2:SUF:2.0.0*.

The results present the regression coefficients and their standard errors in parentheses. Significance levels are marked as follows: ***p < 0.001, * p < 0.5. n = 2,396. Imputed data, M = 73.

## Discussion

This article sets out to analyze how regional differences in educational regulations influence the educational participation of refugee adolescents in Germany. In our study, we looked at four outcome variables that we consider to be important indicators of the further educational trajectories of refugee youth: the duration until school enrollment in Germany, the type of class attended (regular class vs. newcomer class), the type of school attended (other school form vs. *Gymnasium*), as well as age-appropriate schooling. All of these aspects are to a large extent subject to legal requirements that vary, sometimes considerably, from federal state to federal state. Here, often not only the rules themselves, but also the degree of flexibility with which these regulations are implemented vary greatly between federal states. This may lead to different degrees of standardization of school transitions and trajectories of refugee students, which can, in turn, affect the number of opportunities for individual decision-making.

Our analyses show that these legal regulations are indeed significantly related to the educational participation of young refugees. These results appear to be robust to a large extent, even when controlling for additional factors which have been shown to be relevant by previous research on ethnic educational inequality (e.g., Diehl et al., 2016; Will and Homuth, 2020). Family and individual resources seem to play only a minor role in the placement of refugee youth in the German education system. On the other hand, our analyses show that the opportunity structure strongly influences the educational participation of young newly-arrived immigrants. Even if at first glance it may seem a self-evident result that legal regulations for the schooling of new immigrants do in fact influence the integration of newly arrived refugees, we consider these results to be highly relevant. Firstly, it has not yet been possible to demonstrate quantitatively that these regulations are actually statistically related to students' educational participation. Secondly, the results show that the relationships are robust even when other central factors are controlled for, and that a number of factors that are otherwise used to explain participation in education (e.g., student performance, parental education) only have very limited additional explanatory power. This indicates that in the context of lateral entry into the German education system, the scope of action for students and their parents is limited. This makes it all the more important to take a close look at the relationship between legal regulations and the placement of newly-immigrated young people, and to discuss the possible consequences for medium- and long-term integration.

While in some federal states the start of compulsory schooling is very standardized and depends on the arrival in Germany or the federal state itself, in other federal states the start of compulsory schooling is linked to students' assignment to a municipality (see Duration Until School Enrollment). However, assignment to a municipality can depend on many factors, especially the absolute number of refugees to be admitted. Overall, we see that linking compulsory schooling to the assignment to a municipality delays the start of school for one to two months on average. Since most federal states had to process a high number of asylum applications especially in 2015 and 2016, which resulted in a massive administrative backlog, federal states that set time limits for compulsory school provided an advantage for schooling young refugees, even at the peak of refugee migration in Germany. That such differences in education policies for compulsory schooling between federal states are accompanied by actual inequality in waiting times has not been shown by previous research. This result has important policy implications if the goal of educational integration is to be achieved. Considering that the young people have already missed on average more than seven months of schooling, i.e., more than half a school year, due to their flight alone, such further delays prolong the interruption in the educational trajectory, and can lead to further deceleration in learning. The criticism of long waiting times for refugee students applies all the more when we consider that participation in school should not only promote competence development, but also constitutes a possibility for social integration, and enables a structuring of life (McBrien, 2005; de Wal Pastoor, 2015).

With regard to the question of whether refugee adolescents are enrolled at a regular class, or whether they are initially educated in a separate class for new immigrants (see Type of Class Attended), instead of characteristics of social origin and previous school performance, it is the legal regulations for the education of new immigrants that prevail in the respective federal states, that seem to play the most important role. The assignment to a federal state thus determines, to a certain extent, the initial conditions for the further educational trajectory of young refugees. This restrictive framework in the context of assignment leaves little room for individual educational choices. The extent to which the type of schooling is beneficial or detrimental to further educational careers, and whether differences between (e.g., social) groups may emerge in this regard should be the subject of further research based on the results of our study. The forthcoming waves of data from the ReGES panel study, in which the adolescents were followed for two more years of education, will allow research on medium-term effects. In this context, it must be pointed out that in practice the various school strategies often cannot be separated as clearly as the typification of the strategies suggests. In practice, there are many different intermediate forms, which are also used by the respective teachers in different ways to promote learning (Massumi et al., 2015; Korntheuer and Damm, 2020).

Further research is also needed with regard to the effects of age-appropriate enrollment (see Schooling in an Age-Appropriate Grade Level). We see a strong connection between age-appropriate enrollment and the legal regulations in the federal states (see Age-Appropriate Placement in a School Class). Regulations enforcing strong standardization of school entrance are connected to more age-appropriate enrollment. Federal states which do not assign refugee students to a grade level and school type directly when they start school in Germany, but at a later point in time (e.g., 1 year thereafter) show more flexibility regarding age, and seem to consider other aspects like school performance more strongly. The effects this has on educational trajectories, and whether these effects differ for various groups needs to be further analyzed with longitudinal data.

We also observed strong effects of the legal regulations in the individual states with regard to the type of school attended (see Type of School Attended and Access to Different Types of Schools). Regulations that stipulate that new immigrant classes are primarily located at lower school types lead to young refugees being significantly less likely to attend a *Gymnasium* when transitioning to a regular class. In fact, the likelihood of attending a *Gymnasium*, and thus being on a direct track to acquire a university entrance qualification is reduced by 16 percentage points. However, we additionally see a clear positive correlation between parental education and adolescents' self-assessed school performance in their origin country. Building on our results, the next step is to examine the extent to which the ability of students and their parents to influence the type of school attended depends on the degree of standardization of this assignment. It could be assumed that the influence of family and individual resources is greater in states with more flexible approaches toward school assignment.

Although we only consider five federal states, we assume that our results can be applied to other federal states in Germany, provided that the legal regulations in force and the degree of standardization or flexibilization are taken into account. Furthermore, we are convinced that our findings–even if they are based on data from the German context–are informative for other countries, especially for countries with stratified school systems. Our results indicate that the quickest possible access to school *per se*, regardless of the legal situation and accommodation, should be the basis of legal regulations for the schooling of new immigrants. Since in Germany, the allocation of a student to a certain educational track also largely determines their later chances in the labor market (Bol and van de Werfhorst, 2011), we also assume that in stratified education systems the most flexible allocation possible to different types of school should be conducive to medium-term integration into the labor market. In order to assess this more precisely, further studies are required on the educational careers of refugee students in the individual school types, and on their transition to the labor market.

Overall, we observe that the legal regulations strongly pre-structure educational participation and seem to leave little individual leeway in most of the educational decisions made regarding young refugees. It should be noted, however, that the explained variance in our models is rather low. This indicates that other aspects than the ones considered in our analyses also play a role in explaining the participation of new immigrants in the German education system.

In our opinion, there are at least three areas that need to be addressed in this regard by further research. First, the legal regulations at the state level provide the framework in which school administrations and educational specialists in the individual communities and cities act, but there are also differences between the municipalities. On the one hand, this concerns the framework conditions on site (e.g., availability of schools, number of newly arrived immigrants of school age) and, on the other hand, the individual implementation of the requirements. Especially when the legal regulations at the federal state level introduce de-standardization and allow flexibility, the decision-makers in the individual municipalities have significant room for maneuver. In these cases, characteristics of the decision-makers (e.g., experiences in dealing with newly-immigrated students), or the local support structures for refugees (e.g., through volunteers) should also contribute to explaining the educational participation of young refugees. Second, additional individual resources might play a role in the placement of the newly-immigrated youth that we cannot control for in our study. This applies above all to the adolescents' German language skills at the time they start school. In the ReGES study, students' German language skills were measured with objective tests (see Obry et al., 2021). This testing, however, only took place after they had already been enrolled at school. Thus, the influence of German language skills on further educational trajectories can be examined, but not its influence on the placement at the time of school enrollment, which we focus on in our study. Third, while early school enrollment and attending a school that prepares for university are options that educated parents value highly and therefore prefer for their children, the other indicators we examined are less clear-cut. For example, an age-appropriate placement at a school class might be better for the social integration of young people, and with good performance, also enables faster access to vocational training, university, or the job market. On the other hand, for some of the young people who have fled, starting school at an age that is appropriate for them also carries the risk of overloading them, or is at the expense of attending a less demanding school type. By contrast, a deferral in a lower grade level might offer greater opportunities to catch up on learning material. The same applies to schooling in a new immigrant class: attending newcomer classes might enable refugee students to first acquire German language skills and to become familiar with the new school system, but could at the same time, also delay social integration and restrict subsequent educational paths. The fact that the returns linked to these educational decisions can be evaluated differently, and thus make a clear cost-benefit calculation for this educational decision difficult, may lead to the result that we do not see any clear effects of the social origin and adolescents' previous school achievements. It is all the more important to observe the medium and long-term effects these educational decisions have on the educational trajectories and the further participation of young immigrants in society. These results could inform, and possibly serve as decision-making aids for political decisions-makers, teachers, students, and parents alike.

## Data Availability Statement

The dataset was published under the following doi: 10.5157/ReGES:RC2:SUF:2.0.0.

## Ethics Statement

Ethical review and approval was not required for the study on human participants in accordance with the local legislation and institutional requirements. Written informed consent to participate in this study was provided by the participants' legal guardian/next of kin.

## Author Contributions

All authors listed have made a substantial, direct, and intellectual contribution to the work and approved it for publication.

## Funding

The data we used for our research were collected in the framework of the ReGES–Refugees in the German Educational System project. The project was funded by the German Federal Ministry of Education and Research (BMBF) under Grant Number FLUCHT03. The present work took place partly within the framework of the “Educational Trajectories of Refugee Children and Adolescents” project (funded by the BMBF, Grant Number FLUCHT2021) and of the “Educational Integration of Refugee Children and Youth” project (funded by the BMBF, Grant Number 01JG2107). The open access publication of this article was funded by the Open Access Fund of the Leibniz Association.

## Author Disclaimer

The content of this publication is solely the responsibility of the authors.

## Conflict of Interest

The authors declare that the research was conducted in the absence of any commercial or financial relationships that could be construed as a potential conflict of interest.

## Publisher's Note

All claims expressed in this article are solely those of the authors and do not necessarily represent those of their affiliated organizations, or those of the publisher, the editors and the reviewers. Any product that may be evaluated in this article, or claim that may be made by its manufacturer, is not guaranteed or endorsed by the publisher.

## Footnotes

^1^The *Kultusministerkonferenz* aims to achieve standardization, harmonization, and comparability of educational policies between federal states.

^2^This applies to all of the four outcome variables that are the focus of this study (for an overview of the regulations on schooling for refugees in all German federal states, see Massumi et al., 2015).

^3^Massumi (2019) shows for selected immigrant and refugee students in North Rhine-Westphalia that some students are able to diminish waiting times until school enrollment by registering directly at a specific school, even when they have not been officially allocated to a municipality. This seems, however, rather demanding and highly dependent on social networks and schools' willingness and resources to accept students with unsettled resident status.

^4^Baden-Wuerttemberg, North Rhine-Westphalia, Saxony-Anhalt, and Schleswig-Holstein.

^5^It must be mentioned that regulations on newcomers' classes only apply to students who do not speak enough German to follow the lessons. In this respect, new immigrants with sufficient knowledge of German should receive integrated schooling in all the federal states considered. However, since we do not have measures for students' German language skills at the time of enrollment, we cannot empirically test this with the available data.

^6^However, when interpreting the results, it should be noted that even if students with Syrian citizenship are largely refugees, there are also Syrian students who are, for example, descendants of people who immigrated to Germany as highly qualified professionals, or as family members (for North Rhine-Westphalia, Kemper and Reinhardt (2022, p. 5) estimate the proportion of refugees among Syrians in the school system with 66.4%).

^7^This paper uses data from the “Refugees in the German Educational System (ReGES)” project: Refugee Cohort 2–Adolescents, doi: 10.5157/ReGES:SUF:RC2:2.0.0, which was funded by the German Ministry of Education and Research (BMBF) under grant number FLUCHT03, and conducted by the Leibniz Institute for Educational Trajectories (LIfBi).

^8^Unaccompanied minor refugees are therefore not included in the sample. This means that approximately 10% (average for the years 2014-2018; see Bundesamt für Migration und Flüchtlinge (BAMF), 2020; own calculations) of the minors who applied for asylum were not part of the sample. Since most of the unaccompanied minor refugees are male (on average 90%, see Tangermann and Hoffmeyer-Zlotnik, 2018), the proportion of male adolescents among unaccompanied minor refugees is likely to be considerably higher than in our sample.

^9^At the end of 2020, 498,830 refugees were living in North Rhine-Westphalia, followed by Bavaria (213,085), Rhineland-Palatinate (83,605), Saxony (61,510), and Hamburg (51,825). The proportion of refugees in the total population in the respective federal state varies from 1.5% in Saxony and 1.6% in Bavaria, to 2.0% in Rhineland-Palatinate and up to 2.8% in Hamburg and North Rhine-Westphalia (Statistisches Bundesamt, 2021b).

^10^As the ReGES study only included young people who were attending general secondary school, no information can be given about young refugees who do not attend school. Analyses with the data from the IAB-BAMF-SOEP survey of refugees show that 8% of 11–14-year-old refugees and 13% of 15–16-year-old called refugees do not attend school (see de Paiva Lareiro, 2019). Unfortunately, de Paiva Lareiro's study does not examine in more detail whether the refugees are not yet attending school, for example, because they have not lived in Germany long enough, whether they have already left school, or whether other reasons play a role, in these children and adolescents not attending school.

^11^We use LPM instead of a logit model because, compared to LPM, logit models have several disadvantages with hetereoscedastic standard errors (Allison et al., 2020), and estimations with multiply imputed data are not fully implemented for logistic regression in Stata. Nonetheless, to check the robustness of the results, we also calculated all linear probability models as logit models. The results of the logit models show a generally similar picture. The strong correlations between our indicator of educational regulations and the respective dependent variable are also evident and robust, even when controlling for other key variables. The effects of the other variables taken into account do not differ with regard to significance level and the ratio of effect sizes. Only for the analyses of attending a *Gymnasium*, we observe slightly lower significance levels for students whose parents have obtained postsecondary or tertiary education and for students' age in the final model.

^12^Robustness checks with the type of class first attended in Germany show similar results (see Appendix C). However, the effect for students in federal states which almost exclusively assign students to newcomer classes at first (in comparison to the reference group) is only significant in the final model (Model C.4).

^13^We additionally calculated a robustness check with a more conservatively coded dependent variable, which excludes students in comprehensive schools from the analysis (Appendix D). The results confirm the finding that students in federal states which establish newcomer classes at lower school types are indeed less likely to attend *Gymnasium*. The connection between legal regulations and the assignment to a school type is even more pronounced with respect to the effect size. This holds also true for the correlations with parental education, and partially, with previous self-assessed school performance, while the effect size of the adolescents' age at arrival does not change.

^14^But also overall Saxony and Bavaria have a comparatively low high-school graduation rate in relation to the overall population of students (see Statistisches Bundesamt, 2020). This is an indication that the barriers to successfully completing *Gymnasium* in these federal states appear to be high, and not only for newly arrived immigrant students.
