# Supplementary material for: Educational Policies Matter: How Schooling Strategies Influence Refugee Adolescents' School Participation in Lower Secondary Education in Germany
Source: Front Sociol. 2022 Jun 22;7:842543. doi: 10.3389/fsoc.2022.842543 (PMC9256926; doi:10.3389/fsoc.2022.842543)
Supplement: Supplementary file 1 [file Data_Sheet_1.pdf]

Supplementary Material

Appendix A: Overview of dependent and control variables by federal state

	Bavaria		Hamburg		North Rhine-Westphalia		Rhineland-Palatine		Saxony	
	N	%/M	N	%/M	N	%/M	N	%/M	N	%/M
**Dependent variables**										
Duration up to school enrollment (in months)	269	5.58	237	7.28	1,293	7.84	253	6.01	162	6.45
Type of class attended	276	0.34	281	0.60	1,387	0.56	287	0.22	179	0.63
Type of school attended[1]	208	0.05	165	0.29	821	0.22	246	0.17	90	0.07
Age-appropriate placement	274	0.65	278	0.78	1,378	0.66	286	0.74	180	0.61
**Social origin**										
Highest parental ISCED	243		224		1,184		202		164	
No/less than primary education		30.04%		55.36%		32.01%		32.18%		34.15%
Primary education		11.93%		5.36%		10.39%		10.40%		7.93%
Secondary I + II education		33.74%		21.88%		30.32%		32.18%		37.20%
Postsecondary/tertiary education		24.28%		17.41%		27.28%		25.25%		20.73%
Highest ISEI-08 (parents)	233	41.58	216	41.19	1,101	45.08	202	48.50	160	44.15
**Previous educational experiences**										
Self-assessed school performance	243	73.03	226	71.95	1,267	77.91	260	74.24	168	76.51
**Control variables**										
Urbanity	276		281		1,391		287		180	
>500.000 inhabitants		55.80%		100.00%		56.87%		14.29%		79.44%
100.000-500.000 inhabitants		38.04%				33.07%		36.93%		17.22%

<100.000 inhabitants		6.16%				10.06%		48.78%		3.33%
Sex	276	0.45	281	0.44	1,391	0.46	287	0.43	180	0.41
Age (at arrival, in months)	276	162.09	281	162.33	1,391	161.67	287	162.59	180	162.19
Origin country	276		281		1,391		287		180	
Afghanistan		6.88%		29.18%		5.82%		7.32%		8.33%
Iraq		18.84%		11.03%		14.88%		2.09%		12.22%
Syria		64.86%		44.84%		72.68%		77.00%		69.44%
Other		9.42%		14.95%		6.61%		13.59%		10.00%
Resident status	232	0.28	217	0.12	1,254	0.34	233	0.31	166	0.41
Current type of class	276	0.25	281	0.41	1,389	0.41	287	0.14	179	0.50

[1]*Only students who attend a regular class are included in the calculation.*

Source: ReGES data, Refugee Cohort 2–Adolescents, doi:10.5157/ReGES:RC2:SUF:2.0.0
Note: The descriptive statistics for the independent variables are based on the overall sample before imputation and slightly deviate across each set of analyses due to varying number of cases.

Appendix B: Stepwise OLS regression on duration until school enrolment as dependent variable (full model)

	Model B.1	Model B.2	Model B.3	Model B.4
**legal regulations: enrollment…**				
(*ref.: …after assignment to municipality)				
…without delay	-0.156	-0.334	-0.388	-1.169*
	(0.456)	(0.465)	(0.466)	(0.500)
…three months after moving from abroad	-1.855***	-1.914***	-1.993***	-2.282***
	(0.398)	(0.397)	(0.398)	(0.384)
**highest parental ISCED**				
(*ref.: secondary I+II education)				
no/less than primary education		0.932*	0.793*	0.557
		(0.398)	(0.399)	(0.402)
primary education		1.313*	1.268*	1.352*
		(0.645)	(0.644)	(0.619)
postsecondary/tertiary education		0.078	0.125	0.146
		(0.467)	(0.468)	(0.449)
**highest ISEI-08 (parents)**		-0.013	-0.011	-0.011
		(0.008)	(0.008)	(0.008)
**average school performance (origin country)**			-0.019*	-0.021**
			(0.007)	(0.007)
**degree of urbanity**				
(*ref.: >500.000 inhabitants)				
100.000-500.000 inhabitants				-0.333
				(0.323)
<100.000 inhabitants				-0.482
				(0.478)
**time of arrival (in quarters)**				
(*ref: 04/2015)				
01/2014				3.084*
				(1.264)
02/2014				2.574
				(1.381)
03/2014				3.146**
				(1.092)
04/2014				2.168
				(1.183)
01/2015				3.763***
				(1.025)
02/2015				2.093**
				(0.770)
03/2015				1.066**
				(0.399)
01/2016				0.591
				(0.423)
02/2016				-0.763
				(0.516)
03/2016				-2.014***

				(0.540)
04/2016				-3.085***
				(0.447)
01/2017				-3.086***
				(0.428)
02/2017				-3.013***
				(0.480)
03/2017				-5.120***
				(0.659)
04/2017				-5. 285***
				(0.891)
**gender**				
female				0.260
				(0.282)
**age at arrival (in months)**				0.018
				(0.014)
**origin country**				
(*ref.: Syria)				
Afghanistan				0.710
				(0.518)
Iraq				0.393
				(0.407)
other				-0.125
				(0.509)
**resident status**				
insecure resident status				-1.369***
				(0.301)
R^2	0.0080	0.0193	0.0225	0.1054

Source: ReGES data, Refugee Cohort 2–Adolescents, doi:10.5157/ReGES:RC2:SUF:2.0.0

*Note: The analyses are based on data from the study "Refugees in the German Educational System (ReGES)": Refugee Cohort 2–Adolescents, first wave (SUF: 2.0.0). The quarter of arrival were introduced as dummy variables. The results present the regression coefficients and their standard errors in parentheses. Significance levels are marked as follows: ***p<0.001, **p<0.01, p*<0.5. n=2,214. Imputed data, M=73.*

Appendix C: Stepwise linear probability models on type of class attended as dependent variable (first class attended)

	Model C.1	Model C.2	Model C.3	Model C.4
**legal regulations**				
(*ref.: more flexible assignment)				
external differentiation	0.048	0.052	0.053	0.070*
	(0.032)	(0.032)	(0.032)	(0.035)
internal differentiation	-0.308***	-0.306***	-0.305***	-0.267***
	(0.026)	(0.026)	(0.026)	(0.031)
**highest parental ISCED**				
(*ref.: secondary I+II education)				
no/less than primary education		-0.063*	-0.061*	-0.041
		(0.027)	(0.028)	(0.028)
primary education		-0.005	-0.005	-0.009
		(0.041)	(0.041)	(0.041)
postsecondary/tertiary education		-0.086**	-0.087**	-0.088**
		(0.033)	(0.033)	(0.033)
**highest ISEI-08 (parents)**		-0.001	-0.001	-0.001
		(0.001)	(0.001)	(0.001)
**average school performance (origin country)**			0.000	0.000
			(0.001)	(0.001)
**degree of urbanity**				
(*ref.: >500.000 inhabitants)				
100.000-500.000 inhabitants				-0.012
				(0.024)
<100.000 inhabitants				-0.089**
				(0.033)
**gender**				
female				-0.030
				(0.020)
**age at arrival (in months)**				0.004***
				(0.001)
**origin country**				
(*ref.: Syria)				
Afghanistan				-0.109**
				(0.038)
Iraq				-0.002
				(0.031)
other				-0.046
				(0.035)
**resident status**				
insecure resident status				0.038
				(0.023)
R^2	0.0426	0.0512	0.0514	0.0702

Source: ReGES data, Refugee Cohort 2–Adolescents, doi:10.5157/ReGES:RC2:SUF:2.0.0

*Note: The analyses are based on data from the study "Refugees in the German Educational System (ReGES)": Refugee Cohort 2–Adolescents, first wave (SUF: 2.0.0). The results present the regression coefficients and their standard errors in parentheses. Significance levels are marked as follows: ***p<0.001, **<p0.01, p*<0.5. n=2,412. Imputed data, M=73.*

Appendix D: Stepwise linear probability models on access to Gymnasium as dependent variable (conservative coding)

	Model D.1	Model D.2	Model D.3	Model D.4
**legal regulations**				
(*ref.: enrolment more flexbile)				
enrollment at lower school types	-0.217***	-0.213***	-0.207***	-0.217***
	(0.020)	(0.020)	(0.020)	(0.021)
**highest parental ISCED**				
(*ref.: secondary I+II education)				
no/less than primary education		-0.024	-0.006	-0.016
		(0.031)	(0.031)	(0.032)
primary education		-0.034	-0.032	-0.041
		(0.043)	(0.043)	(0.044)
postsecondary/tertiary education		0.120**	0.117**	0.112**
		(0.039)	(0.038)	(0.038)
**highest ISEI-08 (parents)**		0.000	0.000	0.000
		(0.001)	(0.001)	(0.001)
**average school performance (origin country)**			0.003***	0.002***
			(0.001)	(0.001)
**degree of urbanity**				
(*ref.: >500.000 inhabitants)				
100.000-500.000 inhabitants				-0.021
				(0.026)
<100.000 inhabitants				-0.066
				(0.034)
**gender**				
female				0.027
				(0.023)
**age at arrival (in months)**				0.002**
				(0.001)
**origin country**				
(*ref.: Syria)				
Afghanistan				0.017
				(0.040)
Iraq				0.004
				(0.035)
other				-0.041
				(0.038)
**resident status**				
insecure resident status				-0.011
				(0.026)
R^2	0.0486	0.0783	0.0947	0.1064

Source: ReGES data, Refugee Cohort 2–Adolescents, doi:10.5157/ReGES:RC2:SUF:2.0.0

*Note: The analyses are based on data from the study “Refugees in the German Educational System (ReGES)”: Refugee Cohort 2–Adolescents, first wave (SUF: 2.0.0). The results present the regression coefficients and their standard errors in parentheses. Significance levels are marked as follows: ***p<0.001, **<p0.01, p*<0.5. n=1,279. Imputed data, M=73.*
